# Immunologic Control of *Mus musculus* Papillomavirus Type 1

**DOI:** 10.1371/journal.ppat.1005243

**Published:** 2015-10-23

**Authors:** Joshua W. Wang, Rosie Jiang, Shiwen Peng, Yung-Nien Chang, Chien-Fu Hung, Richard B. S. Roden

**Affiliations:** 1 Department of Pathology, The Johns Hopkins University, Baltimore, Maryland, United States of America; 2 Research and Development Department, Papivax LLC, Rockville, Maryland, United States of America; 3 Immunotherapy Division, Papivax Biotech Inc., Taipei, Taiwan, Republic of China; 4 Department of Oncology, The Johns Hopkins University, Baltimore, Maryland, United States of America; 5 Department of Gynecology and Obstetrics, The Johns Hopkins University, Baltimore, Maryland, United States of America; University of North Carolina at Chapel Hill, UNITED STATES

## Abstract

Persistent papillomas developed in ~10% of out-bred immune-competent SKH-1 mice following MusPV1 challenge of their tail, and in a similar fraction the papillomas were transient, suggesting potential as a model. However, papillomas only occurred in BALB/c or C57BL/6 mice depleted of T cells with anti-CD3 antibody, and they completely regressed within 8 weeks after depletion was stopped. Neither CD4+ nor CD8+ T cell depletion alone in BALB/c or C57BL/6 mice was sufficient to permit visible papilloma formation. However, low levels of MusPV1 were sporadically detected by either genomic DNA-specific PCR analysis of local skin swabs or in situ hybridization of the challenge site with an E6/E7 probe. After switching to CD3+ T cell depletion, papillomas appeared upon 14/15 of mice that had been CD4+ T cell depleted throughout the challenge phase, 1/15 of CD8+ T cell depleted mice, and none in mice without any prior T cell depletion. Both control animals and those depleted with CD8-specific antibody generated MusPV1 L1 capsid-specific antibodies, but not those depleted with CD4-specific antibody prior to T cell depletion with CD3 antibody. Thus, normal BALB/c or C57BL/6 mice eliminate the challenge dose, whereas infection is suppressed but not completely cleared if their CD4 or CD8 T cells are depleted, and recrudescence of MusPV1 is much greater in the former following treatment with CD3 antibody, possibly reflecting their failure to generate capsid antibody. Systemic vaccination of C57BL/6 mice with DNA vectors expressing MusPV1 E6 or E7 fused to calreticulin elicits potent CD8 T cell responses and these immunodominant CD8 T cell epitopes were mapped. Adoptive transfer of a MusPV1 E6-specific CD8+ T cell line controlled established MusPV1 infection and papilloma in RAG1-knockout mice. These findings suggest the potential of immunotherapy for HPV-related disease and the importance of host immunogenetics in the outcome of infection.

## Author Summary

While most patients clear human papillomavirus (HPV) infection, some develop persistent papillomas, especially if immunocompromised. Likewise, we find a fraction of outbred SKH-1 mice challenged with *Mus musculus* papillomavirus type 1 (MusPV1/MmuPV1) develop persistent papillomas, whereas most SKH-1 mice, as seen for the inbred C57BL/6 and BALB/c strains, clear the infection. Viral clearance requires both CD4+ and CD8+ T cells, and depletion of either subset permits persistent but subclinical infection. In C57BL/6 mice, CD8+ T cell epitopes were mapped to MusPV1 E6 and E7; however the CD8+ T cell response to E6 dominated and correlated with spontaneous regression. A MusPV1 E6-specific CD8+ T cell line was developed by vaccination and culture *in vitro*, and its systemic administration once was sufficient to effect papilloma clearance in an immunodeficient mouse. Our observations in inbred and outbred mice challenged with MusPV1 suggest promise for immunotherapy to treat HPV-associated disease.

## Introduction

Papillomaviruses (PVs) are 60 nm diameter, non-enveloped, double-stranded DNA viruses that produce papillomas (warts) in a wide variety of organisms, but with strict host tropism [1,2]. Over 120 human papillomavirus (HPV) genotypes have been fully sequenced. HPVs typically cause either mucosal or cutaneous disease [3,4]. The mucosal HPV genotypes comprise mainly genus *Alpha* species, which are further categorized based upon their oncogenicity; ‘low risk’ (lrHPV) *Alpha* types are associated with benign papillomas, whereas the ~15 ‘high risk’ (hrHPV) types have malignant potential [5]. Indeed, hrHPV are present in >99% of cervical cancers and are considered a necessary causal agent [6,7]. The hrHPV, predominantly HPV16, also cause a subset of cancers at other anogenital sites and the oropharynx [8]. In contrast, there are numerous cutaneous HPVs that can cause benign papillomas, such as common, plantar and flat warts, including some of the *Alpha* species (e.g. HPV 2, 27 & 57), as well as *Gamma* species (e.g. HPV4, 65), and *Mu* species (e.g. HPV1) [4]. Cutaneous papillomavirus infection is near ubiquitous and often is not clinically apparent or spontaneously resolves [9,10]. The *Beta* species types HPV5 and HPV8 were first identified in individuals afflicted with the hereditary syndrome epidermodysplasia verruciformis (EV). EV is characterized by extensive and recalcitrant skin warts that can progress to non-melanoma skin cancer (NMSC) in sun exposed areas [11,12]. NMSC associated with *Beta* HPV also occurs in HIV+ or solid organ transplant patients with immune suppression [13–15], but in otherwise healthy individuals their etiologic role in NMSC remains controversial, and they are produce typically asymptomatic cutaneous infection [9].

Demonstration of the etiologic role of HPV16 and HPV18 in 50% and 20% respectively of cervical cancer cases globally has driven the development of prophylactic HPV vaccines. The licensed vaccines, based on L1 virus-like particles (L1-VLP), provide effective but type-restricted protection of naïve patients [16–19]. This type-restriction has driven the licensure of a 9-valent vaccine to broaden coverage to most of the hrHPV common in cervical cancer [20], as well as development of candidate vaccines based upon the minor capsid protein L2, a conserved protective antigen. Unfortunately, given the challenges of global implementation of HPV vaccination, the prevalence of hrHPV infection remains high, especially among older unvaccinated patients in developed countries and of all ages in low resource countries [21,22].

The L1-VLP or L2 vaccines provide no therapeutic benefit for those already infected [23], and there is currently no treatment for established hrHPV infection except repeated screening. Fortunately, half of cervical hrHPV infections in immune competent women, even HPV16 and 18, become undetectable by DNA testing of cervical swabs within a year, consistent with spontaneous immunologic control [24,25]. It is not clear whether immunologic control reflects the complete elimination of the virus or rather the restriction of the virus to small reservoirs in basal cells with potential for recrudescence upon immune senescence, HIV co-infection or active suppression. Indeed, re-activation of previously suppressed infections, rather than acquisition of new infections, has been proposed to drive a second peak in cervical cancer incidence in older peri-menopausal women [26,27]. Likewise immune suppression caused by progressive HIV co-infection or immunosuppressive therapy in transplant patients is associated with dramatically elevated risk for HPV-associated cancer [13–15].

These observations suggest immunotherapy has promise for control of established infections and HPV-associated disease, including cancer [28,29]. The development of such approaches requires a suitable model, but unfortunately HPV does not replicate in animals. The recently discovered *Mus musculus* papillomavirus type 1 (MusPV1/MmuPV1) is an attractive model [30]. However, while MusPV1 can be propagated in nude mice and is skin-trophic [31–33], it is effectively cleared by common inbred laboratory mouse strains, thereby limiting its utility for vaccine pre-clinical studies.

## Results

### Control of MusPV1 by Cellular Immunity

**Fig 1:**
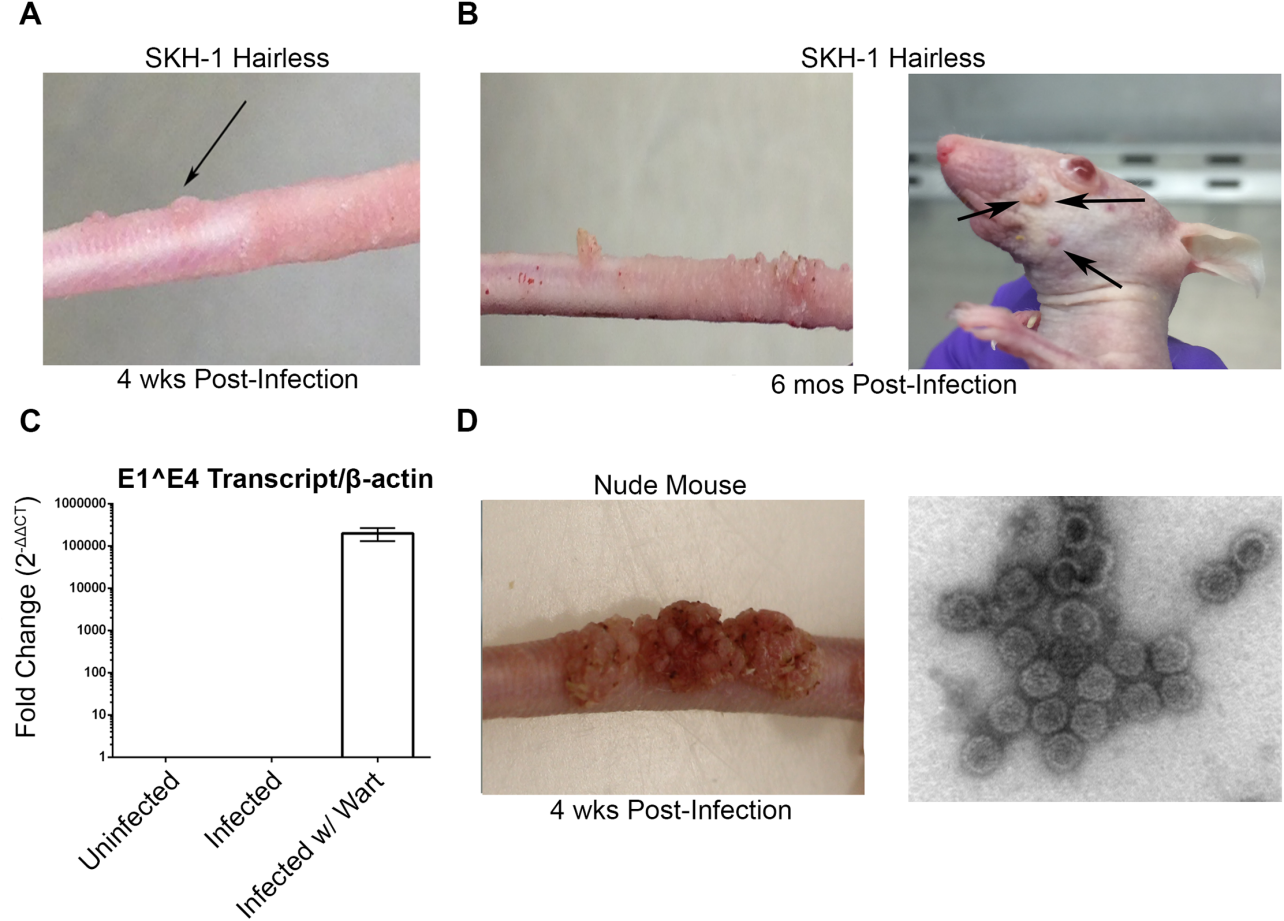
MusPV1 infection and disease in outbred SKH-1 mice and immunocompromised controls. (A) Papilloma formation on the tail of a SKH-1 mouse 4 weeks-post infection, and (B) persisting on the tail over 6 months (left panel) and spreading along the tail and occasionally to the muzzle (right panel). These papillomas were also probed for MusPV1 E1^E4 transcripts suggesting active infection (C). However, the papillomas on SKH-1 mice were not as florid as compared to those on nude mice (D). MusPV1 virions were harvested from the papillomas of nude mice and visualized using negative stain transmission electron microscopy (E).

Consistent with other studies [30–33], MusPV1 infection of immunocompromised mice induced florid papillomas that contained copious amounts of MusPV1 virions (Fig 1A). In contrast, no papillomas were observed in two commonly utilized immune-competent inbred mouse strains, BALB/c and C57BL/6, after challenge. However, upon challenge of female hairless SKH-1 mice, papillomas were observed in 3/20 mice despite normal presence of CD4+ and CD8+ T cells (Fig 1B and 1C) and the apparent immune-competence of this out-bred strain. The papilloma on 2/3 of these mice subsequently regressed leaving a single mouse with persistent disease even after 6 months post infection (Fig 1C) with papillomas spreading to its muzzle (Fig 1E) that also express MusPV1 E1^E4 transcripts (Fig 1G). A MusPV1 L1-specific neutralizing antibody response was observed in serum of all SKH-1 mice at 5 weeks post-challenge (Fig 1F), demonstrating all were exposed to MusPV1.

Whereas contemporaneously challenged nude mice all developed papillomas (Fig 1D), in a repeat study with 20 male and 20 female SKH-1 mice, 9/40 mice developed papillomas (4 female, 5 male). For 2 SKH-1 mice of each gender their papillomas regressed within 3–5 weeks. However, even after 6 months, no regression was observed in the remaining 5. Rather their papillomas continued to expand slowly at the challenge site. Although spreading of papillomas outside of the challenge site did eventually occur in a minority of SKH-1 mice, they remained smaller than the papilloma seen in nude mice (Fig 1D vs. 1E).

### CD4+ and CD8+ T Cells Contribute to Control Papilloma Formation

C57BL/6 mice deficient in CD4, CD40 Ligand, or Type I interferon were challenged (Table 1). In addition, antibody mediated depletion of either CD4+ or CD8+ T cells was performed in immunocompetent BALB/c mice or C57BL/6 mice, prior to and after challenge with MusPV1. Surprisingly, no papillomas were observed within 6 months of challenge in any of these depleted or knock-out mouse groups (Table 1) despite the consistent induction of papilloma on contemporaneously challenged nude mice. In contrast, depletion of CD3+ T cells in both BALB/c and C57BL/6 mice permitted florid growth of papillomas (S1 Fig). When administration of the CD3-specific antibody was stopped, the papillomas remained during the first 4 weeks, presumably due to the delay in reconstitution of the T cell population and its activation, but then began to shrink during weeks 5–6, and were no longer visible by week 10 (S2 Fig), suggesting T cell control [32].

**Table 1:** Papilloma formation following MusPV1 challenge of mice with respect to strain, genetic knock out and immune depletion

Mouse Strain	Immune treatment	Immune Status	Fraction with Papilloma (n of n)
Nude Mice Athymic NCr-nu/nu	N/A	No T cells	100% (50/50)
SCID mice NCI SCID/NCr (BALB/c)	N/A	No B- and T cells	100% (50/50)
BALB/c	Anti-CD3	Depleted total T cells	100% (15/15)
BALB/c	Anti-CD4	Depleted CD4+ T cells	0% (0/15)
BALB/c	Anti-CD8	Depleted CD8+ T cells	0% (0/15)
BALB/c	N/A	Intact inbred immunity	0% (0/15)
C57BL/6	Anti-CD3	Depleted total T cells	100% (15/15)
C57BL/6	Anti-CD4	Depleted CD4+ T cells	0% (0/5)
C57BL/6	Anti-CD8	Depleted CD8+ T cells	0% (0/5)
C57BL/6	N/A	Intact inbred Immunity	0% (0/15)
C57BL/6	CD4-Knockout	No CD4+ T cells	0% (0/5)
C57BL/6	CD40 Ligand- Knockout	No T help	0% (0/2)
C57BL/6	Type-1 Interferon Knock-out	Decreased Anti-viral innate immunity	0% (0/2)
Rag1 KO (C57BL/6)	Rag1 deficient	No B- and T cells	100% (10/10)
SKH-1 Hairless mice (Outbred)	N/A	Intact outbred Immunity	20% (n = 12/60) (6 persistent, 6 regressor)

**Fig 2:**
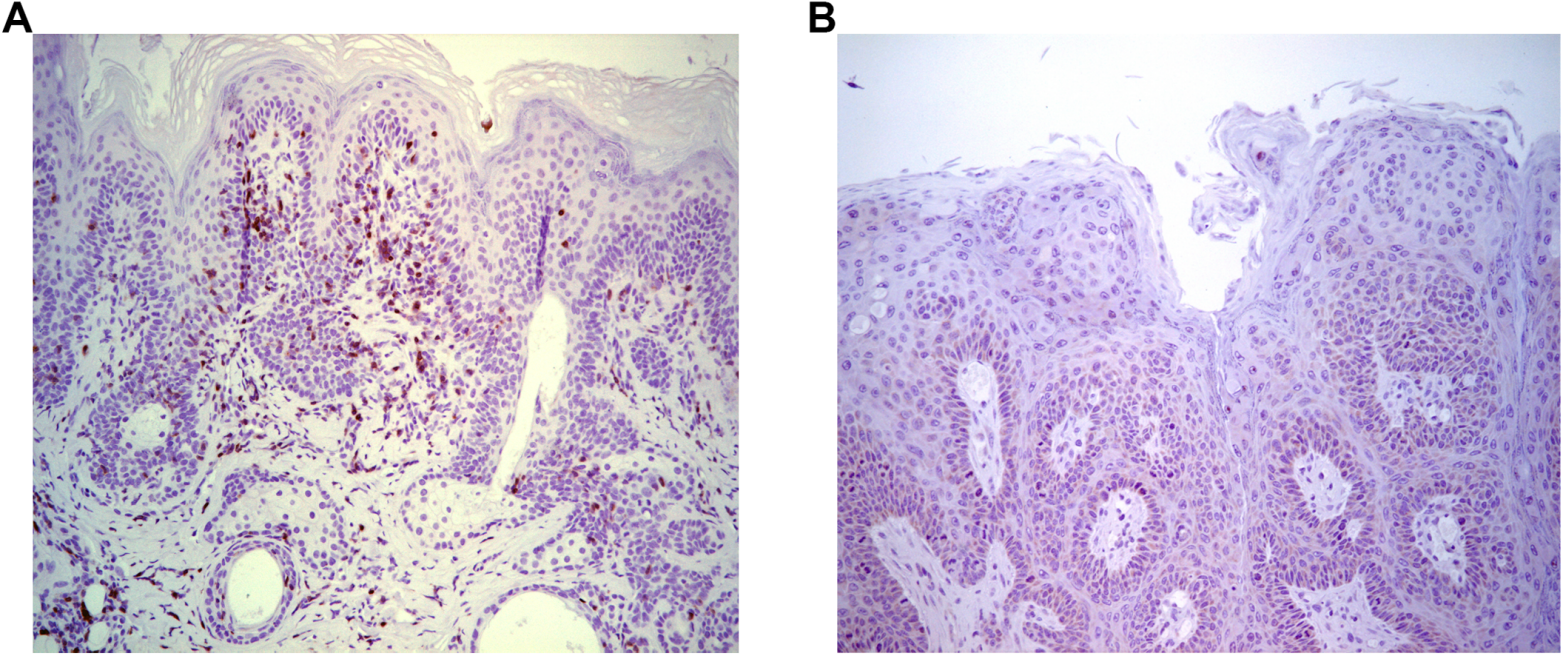
Infiltration of T cells into papilloma site associated with MusPV1 papilloma regression. Immunohistochemical analysis using CD3-specific antibody of a papilloma on SCID mice that had received BALB/c splenocytes by 5 weeks post adoptive transfer and initiated papilloma regression (A) versus control SCID (BALB/c background) with progressive papilloma (B).

To further assess T cell control, adoptive transfer 5x10^6^ splenocytes from naïve BALB/c mice into papilloma-bearing SCID mice of BALB/c background (n = 3) was tested. This triggered slow (~3 months) regression, possibly reflecting the low amount of immune cells transferred, delayed engraftment, activation and/or expansion. Immunohistochemical staining with CD3-specific antibody suggested an infiltration of the regressing papilloma with T cells (Fig 2A), which was absent from control SCID mice (Fig 2B). These results suggest specific immune cells were able to traffic to the site of MusPV1 infection/disease and effect papilloma clearance. These results were also consistent with a recent study by Handisurya *et al* [32] who showed MusPV1 infection resulted in the recruitment of CD4+ and CD8+ T cells, to the lesion.

### Reactivation of Asymptomatic Infection by Immune-Suppression

HPV-associated disease is more recalcitrant and progressive in both solid organ-transplant recipients (OTR) and HIV+ patients with low CD4^+^ T cell counts [2]. Maglennon *et al* also showed in immune competent rabbits persistence of Rabbit Oral Papillomavirus (ROPV) genome in the basal epithelium at the site of infection following papilloma regression [34,35]. Therefore in C57BL/6 and BALB/c mice that were depleted of either CD4+ or CD8+ T cells and failed to develop papilloma post MusPV1 challenge, we tested for the presence of MusPV1 genomic DNA 5 weeks post-challenge via qPCR analysis of tail swabs. Normalization for sampling was provided by measuring mouse β-actin DNA (S3 Fig). The levels of MusPV1 genomic DNA detected in tail swabs of either CD4+ or CD8+ T cell depleted mice were >10^4^-fold lower than for nude mice with papilloma, and were not significantly different from non-depleted mice (S3 Fig). However, it was not clear whether the low level of viral DNA detected by qPCR in these tail swabs reflected a very low level of persistent infection or remnants of the challenge inoculum.

**Fig 3:**
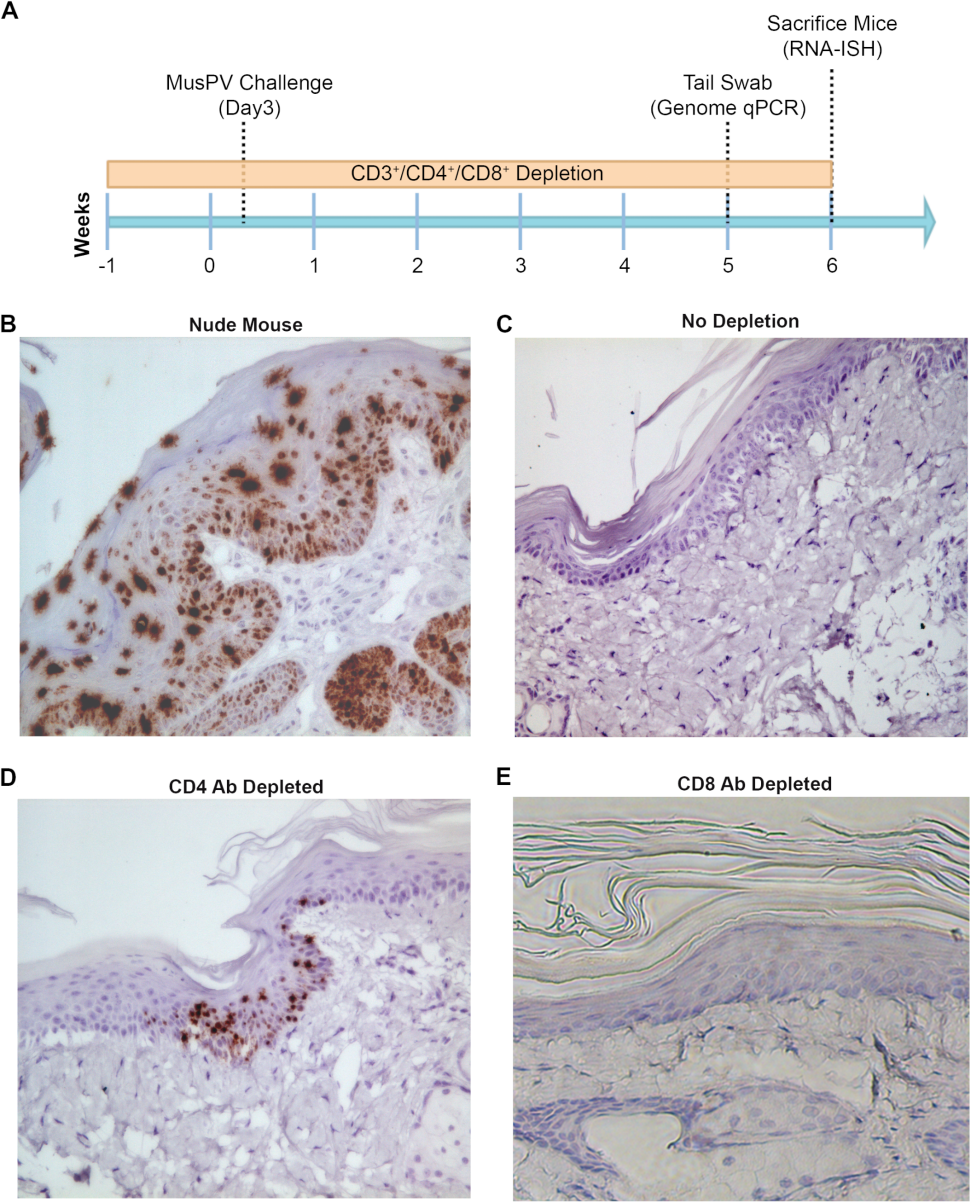
Detection of persistent infection of MusPV1 despite absence of papilloma. Schedules of T cell depletion using anti-CD4/CD8/CD3 monoclonal antibody and the timing of MusPV1 challenge and virus measurements in BALB/c mice. Challenge of nude mice was used as a positive control (A). MusPV1 E6/E7 transcripts were detected in wart bearing nude mice (B) but not wildtype infected mice (no depletion) (C). Low levels of MusPV1 E6/E7 transcripts were present in CD4+ T cell-depleted mice (D), but not those depleted of CD8+ T cells (E).

Subclinical or “latent” papillomavirus infection has been proposed to reside in the basal cell layer in other animal papillomavirus models [34,35]. Therefore we utilized a highly sensitive chromogenic RNA *in situ* hybridization technique (RNAscope) with a probe targeting E6/E7 transcripts on FFPE tail specimens of mice either depleted of CD4+ and CD8+ T cells prior to challenge. High levels of signal were present in papillomas of nude or CD3+ T cell depleted mice, mostly concentrated in the lower epithelial levels (Fig 3B). However, we also noted the presence of strong staining in the upper strata of the epithelium in papilloma-bearing mouse samples, most likely due to the weak hybridization with MusPV1 genomic DNA in cells producing undergoing vegetative replication and production of virus (Fig 3B). Importantly however, this was not seen in tail specimens from CD4+ T cell depleted mice. Rather, staining in the lower levels of the epithelium was only very sporadically observed at 6 weeks post challenge (Fig 3D). However, evidence of MusPV1 *E6/E7* transcripts was not seen in the basal layer in CD8+ T cell depleted mice (Fig 3E) despite detection of viral genomic DNA in swabs by qPCR (S3 Fig). Indeed their histological staining pattern was indistinguishable from wildtype BALB/c or control tail specimens in which no trace of MusPV1 *E6/E7* transcript was detected in 10 tails (Fig 3C).

**Fig 4:**
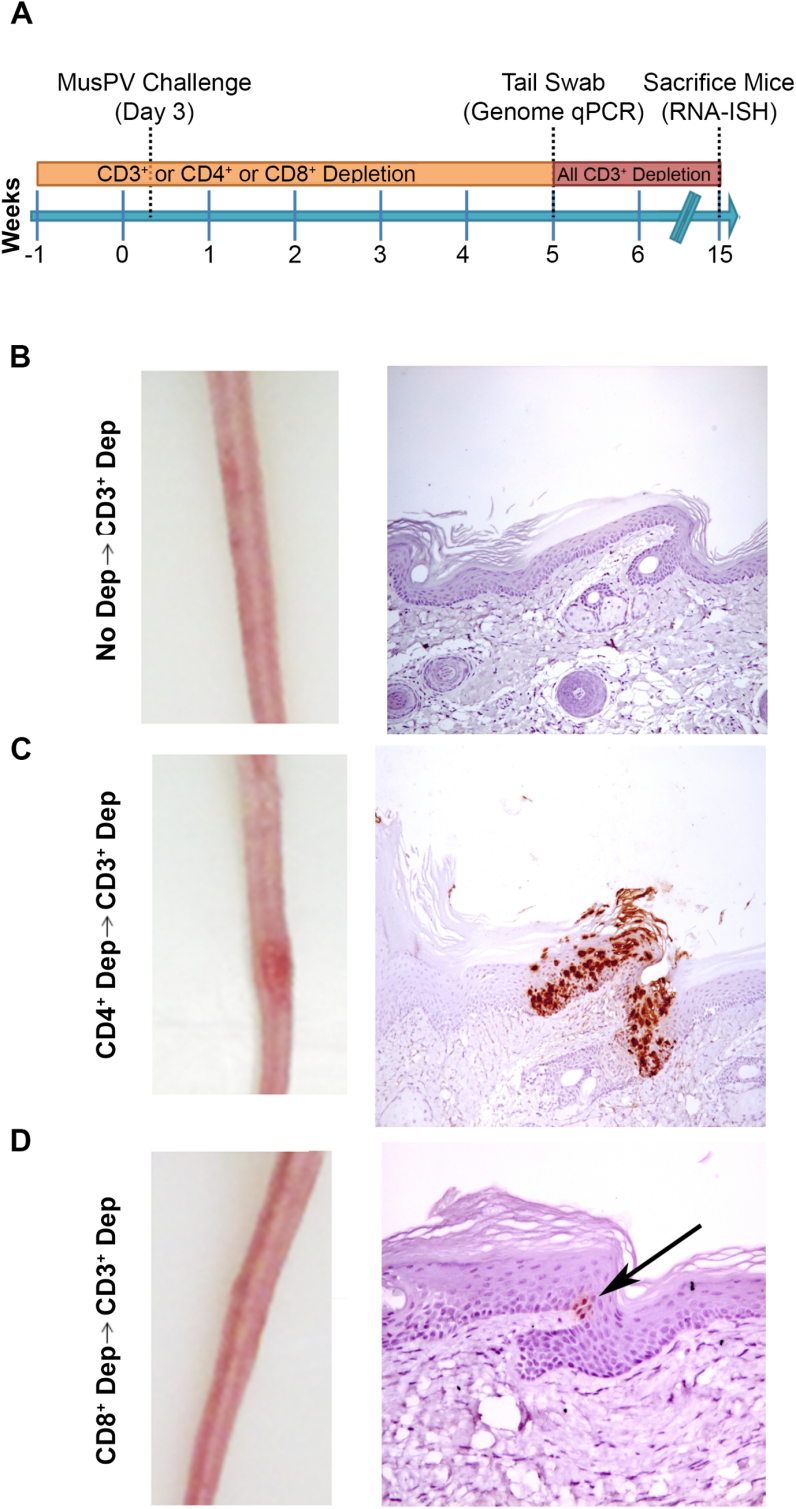
Re-activation of MusPV1 infection. (A) Schema of experiment in BALB/c mice, including schedule of T cell depletion using monoclonal antibodies to CD3, CD4 or CD8 before MusPV1 challenge to the tail. At five weeks post challenge, all mice were switched to treatment with CD3 antibody for ten weeks. Representative gross tail images taken 15 weeks post challenge (left panel) in mice that received no depletion during first 5 weeks (B), or T cell subset depletion using anti-CD4 antibody during first 5 weeks (C), or anti-CD8 antibody during first 5 weeks (D). The right panels of B-D show representative histologic images of tail sections collected after the additional 10 weeks of T cell subset depletion with CD3 antibody for groups B-D and upon *in situ* hybridization for MusPV1 E6/E7 transcripts and hematoxylin staining.

As an approach to detect potentially transcriptionally-inactive MusPV1 virus in the mice that might not be detected by RNA in situ hybridization with *E6/E7* probe, we sought to reactivate viral reservoirs by depleting all (CD3+) T cells. Thus, following CD4+ T cell, CD8+ T cell or mock depletion during and for 5 weeks post MusPV1 challenge, all mice were then switched to anti-CD3 antibody administration for a further 10 weeks to allow for re-activation of any persistent MusPV1 infection (as summarized in Fig 4A). Cages were changed each week to limit potential for fomite contamination or carriage of virus. Following 10 weeks of CD3+ T cell depletion, 14/15 of the initially CD4+ T cell depleted mice, but only 1/15 CD8+ T cell depleted mice and none of the initially mock-depleted mice grew papillomas (Fig 4B–4D). These findings are generally in accord with both the qPCR results (S3 Fig) and the MusPV1 *E6/E7* in situ hybridization analysis (Fig 4), although infrequently low level signal was detected in the basal epithelia of challenged CD8+ T cell depleted mice (1 small patch in 2 of 10 tails examined) that were subsequently depleted with CD3 antibody (Fig 4D).

### CD8 T Cell Recognition of MusPV1 E6 and E7 in C57BL/6

Our results are consistent with other studies suggesting that T cells are critical for MusPV1-papilloma control [31–33]. However, the relevant epitopes have not been characterized. Therefore, to characterize CD8+ T cell epitopes of MusPV1 E6 and E7, each viral protein and a fusion protein of MusPV1 E6, E7 and L2 (aa11-200) were fused with CRT in DNA vaccines (CRT/mE6, CRT/mE7 or CRT/mE6E7L2 respectively). This design was chosen because we have previously shown that DNA vaccines based on fusion of human calreticulin (CRT), an endoplasmic reticulum resident protein that enhances antigen presentation via MHC class I of linked epitopes, with HPV16 E6 and E7 antigens elicits potent HPV16-specific CD8+ T cell and antitumor responses in mice, and when fused with L2 11–200 can induce neutralizing antibodies [36–38].

**Fig 5:**
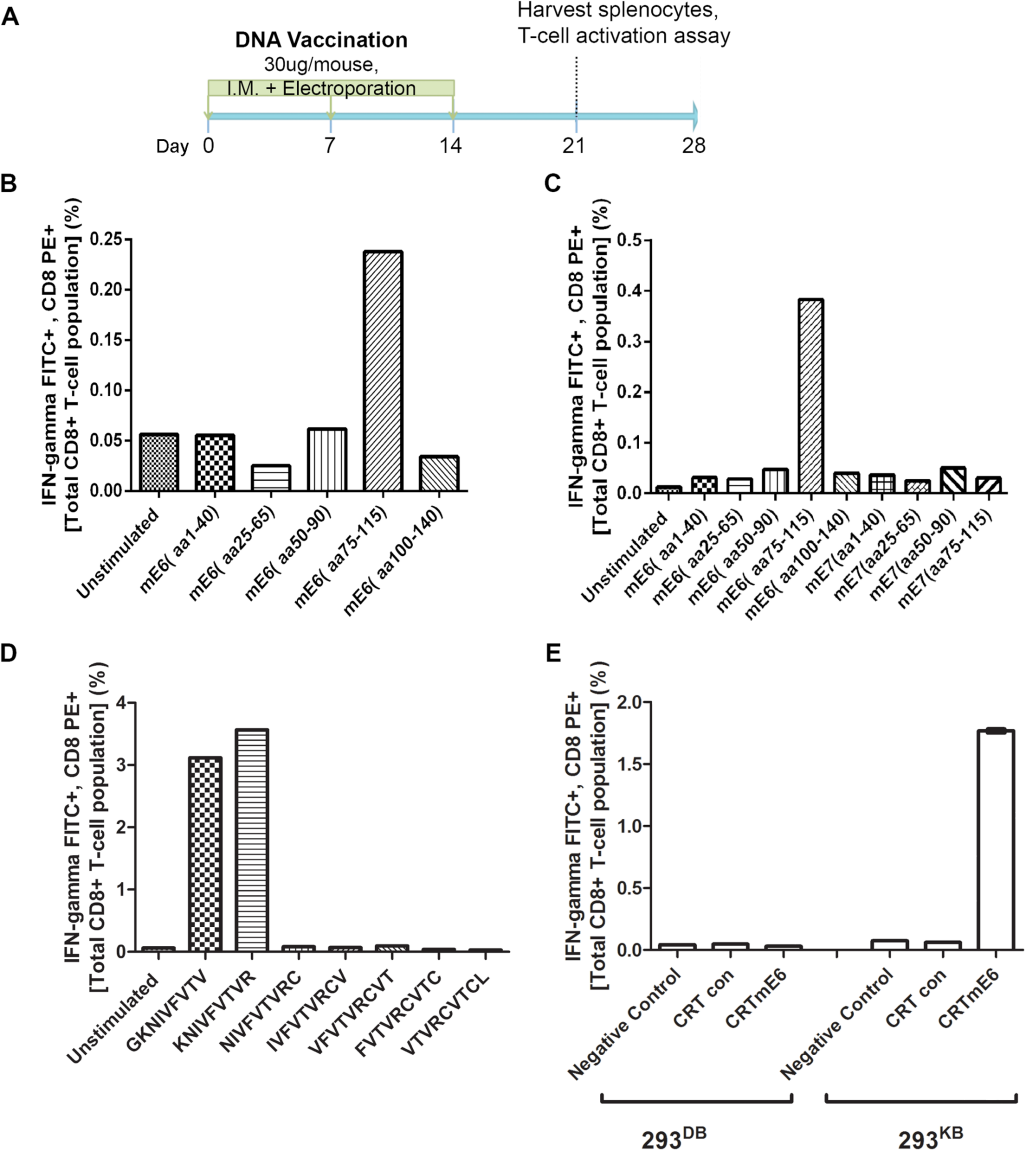
Intracellular cytokine staining with flow cytometry analysis reveals immunodominant CD8+ T cell epitope in MusPV1 E6 and its MHC class I restriction. Schematic of immunization schedule of C57BL/6 mice with DNA vaccines expressing CRT (calrecticulin) fused to different MusPV1 proteins and subsequent harvest of splenocytes (A). Bar graph of flow cytometry data after intracellular cytokine staining of splenocytes for interferon-γ and CD8 after harvest from CRT/mE6-vaccinated mice and stimulation with mE6 peptide library pools (B). Bar graph of flow cytometric analysis of intracellular cytokine staining of splenocytes for interferon-γ and CD8 after harvest from CRT/mE6E7L2-vaccinated mice and stimulation with both mE6 and mE7 peptide library pools (C). Bar graph of flow cytometry data after intracellular cytokine staining of splenocytes for interferon-γ and CD8 after harvest from CRT/mE6 and stimulated with candidate 9mer peptides to map the MHC class I epitopes of MusPV1 E6 (D). Bar graph of flow cytometry analysis showing percentages of interferon-γ expressing mE7-specific CD8+ T cells after co-incubation with 293-K^b^ or 293-K^d^ cells that were transfected with either CRT/mE6 or CRT-alone plasmid (E). All data was repeated and representative images provided.

BALB/c and C57BL/6 (n = 5 each) were vaccinated weekly with CRT/mE6, CRT/mE7 or CRT/mE6E7L2 for 3 weeks. A week after the last vaccination, splenocytes were harvested and T cell activation assays were performed (Fig 5A). For initial screening, we co-cultured the C57BL/6 and BALB/c-derived splenocytes respectively with either 293^DbKb^ cells or CT26 cells (which express the relevant murine MHC class I molecules) that were transfected with CRT-alone, CRT/mE6 or CRT/mE7. A strong mE6-specific T cell response was observed in splenocytes harvested from C57BL/6 mice vaccinated with CRT/mE6, whereas there was no immune response detected against mE7 (S4 Fig). Surprisingly, no detectable CD8+ T cell immune responses against mE6 or mE7 were observed in the BALB/c background (S4 Fig).

To increase sensitivity, we repeated the CD8+ T cell activation assays using 20mer over-lapping peptide libraries derived from MusPV1 E6 and E7 amino acid sequences for stimulation. A strong mE6-specific CD8+ T cell response was detected in splenocytes of C57BL/6 mice vaccinated with CRT/mE6 (Fig 5B) or CRT/mE6E7L2 (Fig 5C). A weaker mE7-specific CD8+ T cell response could be detected in splenocytes of mice vaccinated with CRT/mE7 (S5 Fig). Interestingly the mE7-specific CD8+ T cell response was still not detected in the splenocytes of CRT/mE6E7L2 vaccinated mice (Fig 5C), suggesting immune-dominance of mE6 over mE7 (Fig 5C). However, despite the known increased sensitivity of using peptide pools, no mE6 or mE7 T cell responses could be detected in the splenocytes of vaccinated BALB/c mice despite using these peptide libraries (S4 Fig). This suggests that the cellular response mediating papilloma regression in BALB/c mice is possibly directed towards other viral proteins.

### In C57BL/6 Mice the Identified MHC Class I Epitopes of MusPV1 E6 and E7 are both H-2K^b^ Restricted

These initial results led to a focus on C57BL/6 mice. The use of pools of 20mer overlapping mE6 and mE7 peptides suggested their dominant epitopes lie within residues 75–115 of mE6 (Fig 5B and 5C) and within residues 50–90 of mE7 (S5 Fig). To determine the specific epitope sequence, we designed several 9mer epitope peptide candidates that overlap by one amino acid for mE6 within the 89–104 region and mE7 within residues 66–80. T cell activation assays were then performed using splenocytes from either CRT/mE6 or CRT/mE7 vaccinated mice and stimulation with their respective 9mer candidates. The immunodominant epitope of MusPV1 E6 was localized at amino acids 90–99 (KNIVFVTVR) (Fig 5D) and in MusPV1 E7 at 69–77 (VLRFIIVTG) (S5 Fig). Following elucidation of these epitopes for C57BL/6, we examined which H-2^b^ MHC class I molecule presents these epitopes by co-culture of splenocytes from either CRT/mE6 or CRT/mE7 vaccinated mice with either 293^Kb^ or 293^Db^ cells transfected with their respective viral antigen or CRT alone as a control. Fig 5E and S5 Fig show that the MHC class I epitope of mE6 and mE7 are both H-2^Kb^ restricted in the C57BL/6 background.

### Spontaneous mE6-specific CD8 T Cell Response Correlates with MusPV1 Papilloma Clearance in C57BL/6 Mice

It is possible that epitopes in other MusPV1 proteins are recognized by CD8 T cells during natural regression. Further, given the negative results for BALB/c in our peptide library studies with mE6 and mE7 (S4 Fig), this seemed even more possible. To assess this possibility, CD3+ T cell depletion was stopped in groups of 5 C57BL/6 and 5 BALB/c mice bearing florid MusPV1 papillomas. Once the papillomas had completely regressed, splenocytes were harvested and pooled for each strain. Splenocytes of C57BL/6 and BALB/c mice were incubated with 293^DbKb^ or CT26 cells respectively that had been transfected with expression vectors for CRT alone, or CRT fused to MusPV1 E1, E2, E4, E6, E7, L1 or L2. The C57BL/6 splenocytes were also stimulated directly using the MusPV1 E6 a.a. 90–99 (KNIVFVTVR) and E7 a.a. 69–77 (VLRFIIVTG) peptide epitopes. Only a mE6-specific CD8+ T cell response could be clearly detected in the splenocytes of C57BL/6 mice that had spontaneously cleared their papilloma although, interestingly, there was also a weak L1 T cell response (S6 Fig). However, no MusPV1 E7-specific CD8 T cell response was detected in these C57BL/6 mice, even when their splenocytes were directly stimulated with the mE7 peptide (S6 Fig). Likewise, no response against the other MusPV1 full length viral proteins except L1 was detected. Unexpectedly, no CD8+ T cell responses were detected for BALB/c again (S6 Fig), suggesting a potential lack of sensitivity in the assay due to weak antigen expression or presentation. However, these findings suggest E6 is the dominant antigen in C57BL/6 mice that is presented naturally and is targeted by a CD8+ T cell response that correlates with clearance of MusPV1 papillomas.

### Adoptive Transfer of E6-specific T Cells Prevents the Development of Papilloma in MusPV1-Infected Immunodeficient Mice

**Fig 6:**
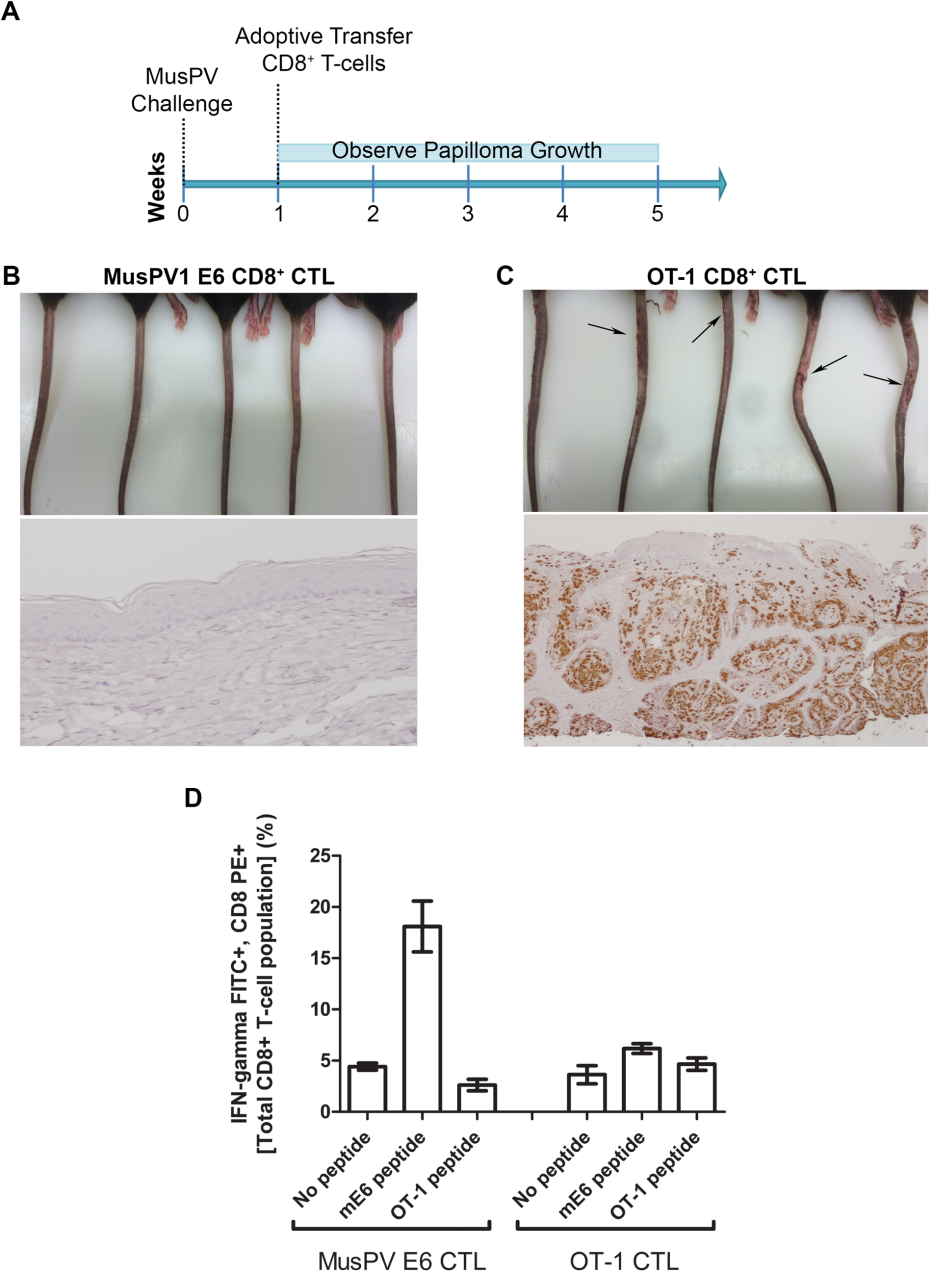
Adoptive transfer of E6-specific CD8+ cytotoxic T cell line one week after MusPV1 challenge prevents papilloma formation in immunodeficient mice. Schematic of study in RAG1 knock-out mice that received adoptive transfer of either 5x10^6^ CD8+ MusPVE6-specific T cell line, or 5x10^6^ OT-1 cells one week after MusPV1 challenge (A). Photographs and tail sections stained for MusPV1 *E6/E7* transcripts of RAG1 knock-out mice 5 weeks post-adoptive transfer with either the MusPV1 E6-specific CD8+ T cell line (B) or OT-1 cells (C). Detection of MusPV1 E6 and OVA peptide specific CD8+ T cells in the spleens of RAG1 knock-out mice 5 weeks post adoptive transfer (D).

The CRT-mE6 or CRT-mE6E7L2 vaccine elicited an E6 a.a. 90–99 specific CD8+ cytotoxic effector T cell response similar to that present in C57BL/6 mice after spontaneous regression of MusPV1 papilloma in unvaccinated mice. To examine whether this response is sufficient to clear an established infection and thereby prevent subsequent papilloma formation, we expanded for adoptive transfer experiments a MusPV1 E6 a.a. 90–99 specific CD8 cytotoxic T cell line *in vitro* (S7 Fig) from the splenocytes of C57BL/6 mice previously vaccinated with CRT-mE6 DNA three times by *in vivo* electroporation. Then, RAG1-KO C57BL/6 mice (n = 10) were first challenged with MusPV1 using a dose sufficient to produce small papillomas within 3–5 weeks. A week after challenge, 5 of the mice were administered the MusPV1 E6-specific CD8+ T cell line *i*.*v*. (5×10^6^ cells), and 5 received 5×10^6^ OT-1 cells, an ovalbumin (OVA)-specific CD8+ T cell line (Fig 6A). By week 4, the tails of the mice that received the MusPV1 E6-specific CD8+ T cells had healed and lacked papillomas (Fig 6B). Conversely, the tail of each mouse treated with OT-1 cells had developed small papillomas by five weeks post-challenge (Fig 6C). Further analysis of these tails for MusPV1 E6/E7 transcript by in situ hybridization revealed strong staining in the papilloma of the mice treated with OT-1 cells after MusPV1 challenge (Fig 6C), whereas the tails of mice treated with adoptive transfer of the MusPV1 E6-specific CD8+ T cell line were devoid of signal, suggestive of viral clearance or profound suppression of early transcription (Fig 6B).

### Clearance of Established Papillomas and Suppression of MusPV1 Infection in Immunodeficient Mice by Adoptive Transfer of E6-Specific CD8+ T Cell Line

**Fig 7:**
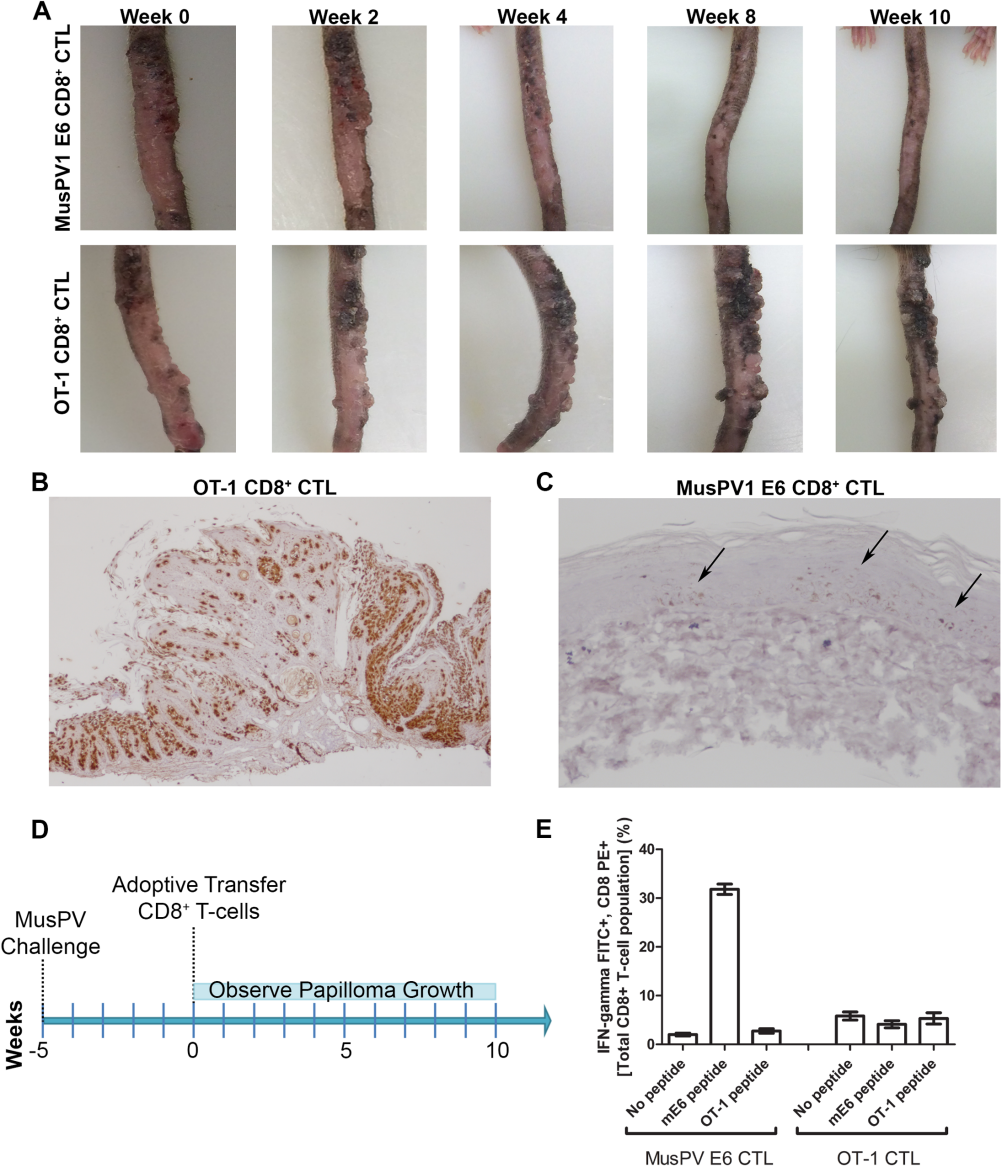
Control of established papilloma by adoptive transfer of MusPVE6-specific CD8+ T cell line. RAG1 knockout mice, n = 4 per group, were challenged with MusPV and papillomas were allowed to grow. After 5 weeks, the papilloma-bearing RAG1 knockout mice received by adoptive transfer either 5x10^6^ CD8+ MusPVE6-specific T cell line or 5x10^6^ CD8+ OT-1 specific T cells. Mice were photographed every week thereafter for 10 weeks until the tails were harvested, sectioned and processed for MusPV1 E6/E7 in situ hybridization by RNAscope and hematoxylin staining. Photographs of one representative mouse from each treatment group are shown over the time 10 weeks post adoptive transfer (A). Analysis of MusPV1 E6/E7 transcription in representative tails harvested from mice that had 10 weeks prior received by adoptive transfer either MusPV E6-specific CD8+ T cells (B) or OT-1 T cells (C). Schematic of MusPV infection of RAG1 knock-out mice and subsequent adoptive transfer of MusPVE6 T-cell line or OT-1 cells as a control (D). Spleens were harvested from mice that had 10 weeks prior received by adoptive transfer either MusPV E6-specific CD8+ T cells or OT-1 T cells. A flow cytometric analysis was performed after intracellular cytokine staining of these splenocytes for interferon-γ and CD8 after stimulation with either mE6 or OVA peptide (E).

To examine impact on established disease, 8 RAG1-KO C57BL/6 mice were challenged with MusPV1 and maintained for 5 weeks until all had fulminant papillomatosis. Subsequently, four mice were administered *i*.*v*. 5x10^6^ CD8+ T cells specific for MusPV1 E6, while the other four received 5x10^6^ OT-1 cells (Summarized in Fig 7D). The papillomas on those mice treated with OT-1 cells continued to grow (Fig 7A, bottom panel), whereas the papillomas on mice treated with the MusPV1 E6-specific CD8 T cell line stabilized for 3 weeks, shrank significantly by weeks 6–8 (Fig 7A, top panel) and were not apparent by 10 weeks post treatment. To assess viral control, their tails were analyzed for MusPV1 *E6/E7* transcript by in situ hybridization. Strong staining was seen in the papilloma of the mice treated with OT-1 cells (Fig 7B). By contrast only in some sections of the tails of the mice that received the MusPV1 E6 specific CD8+ T cell line, were traces of MusPV1 *E6/E7* transcript detected (Fig 7C). These findings suggest MusPV1 E6-specific CD8 T cells can, upon adoptive transfer *i*.*v*., home to papilloma, mediate clinical remission and strongly suppress viral transcription by 10 weeks.

### Expansion of E6-Specific CD8+ T Cells in MusPV1 Infected Mice to Mediate Clearance of Persistent Infection or Disease

Following completion of the adoptive transfer studies (Figs 6 and 7), splenocytes from each group were pooled and stimulated with either MusPV1 E6 or OT-1 peptide. Intracellular cytokine staining for IFN-γ revealed a CD8+ T cell population specific for MusPV1 E6 in the spleens of mice that were protected from (Fig 6E) or cleared established papilloma (Fig 7E). Conversely, no OVA peptide-specific CD8+ T cell response was detected in the spleens of mice that had received OT-1 specific CD8+ T cells, suggesting loss in the absence of cognate antigen. Conversely, the presence of papilloma, or even subclinical infection, provided sufficient antigen to support ongoing proliferation of adoptively transferred MusPV1 E6-specific CD8+ T cells.

## Discussion

HPV-associated diseases are more recalcitrant and progressive in patients with an immune system compromised by hereditary genetics (e.g. EDV or WHIM syndrome), drugs to prevent organ transplant rejection or co-infection with HIV [9,12]. In contrast, most immune-competent individuals clear HPV infection, often without apparent disease, whereas others develop papillomas that are either slowly cleared, or in some persist and/or progress [39]. In patients that clear HPV disease, it is unclear whether there is viral latency and/or the establishment of asymptomic chronic HPV infection which the host immune system constantly suppresses [40]. Here we utilize a recently described laboratory mouse papillomavirus (MusPV1) to serve as a model to further understand interactions between papillomavirus infection and host immunity. While there are other animal models of PV infection in which to investigate this, their high costs and technical limitations have limited progress [34]. Conversely, a papillomavirus model in laboratory mice, with the ready availability of many immunological reagents and genetic knock outs, opens up many opportunities for studies of immune control [30–33].

Studies previously in both rabbits and dogs using rabbit oral papillomavirus (ROPV) and canine oral papillomavirus (COPV) respectively showed evidence of asymptomatic infection [41–43]. In a more recent study, Maglennon *et al* observed long term persistence of ROPV DNA after clearance of visible disease and that immunosuppression with cyclosporine and dexamethasone could reactivate persistent asymptomatic papillomavirus infection to elicit papilloma [34,35]. In line with such findings, we detected very low level viral DNA in tail swabs but no MusPV1 viral *E6/E7* transcripts. However, we failed to recover papilloma after antibody depletion of CD3+ T cells after challenging healthy mice. Nevertheless, when either CD4+ or CD8+ T cells are depleted individually, small reservoirs of infected cells remain in the challenged epithelia (Fig 3) that can potentially be reactivated upon antibody depletion of CD3+ T cells (Fig 4). This was markedly more effective in the CD4+ T cell depleted depleted mice. This is reminiscent of recalcitrant HPV infection in HIV+ individuals, including increased severity of disease and higher rates of HPV-associated cancer as CD4+ T cell counts drop and AIDS progresses [13,44], although there are clearly many of additional immune deficits in these patients. Importantly, these findings support the hypothesis that variations or defects in host immunity are associated with papillomavirus disease and viral pathogenicity [40,41,45].

Our results are consistent with recent reports that CD8 knockout mice of C57BL/6 background control MusPV1 infection [32]. CD8+ T cell depleted mice, unlike the CD4+ T cell depleted mice, were able to mount a neutralizing antibody response (S8 Fig), that may have restrained the re-emergence of disease in the former after CD3+ T cell-depletion. While the neutralizing antibodies are not expected to act directly on infected cells, their presence can block the spread and reduce the load of virus within the host if it re-emerges from viral reservoirs [46]. This was also suggested in studies in COPV whereby spontaneous regressing papillomas were associated with the induction systemic antibodies that could neutralize COPV and potentially prevent spread of intra-oral infection [47]. Furthermore, the presence of systemic L1-specific neutralizing anitbodies is sufficient to confer full protection against MusPV1 challenge as demonstrated recently passive transfer studies of L1-specific antibodies to nude mice [32]. Furthermore, in the *Mastomys natalensis* papillomavirus (MnPV) model, L1-VLP vaccination prevents skin tumor development and progression after immunosuppression with cyclosporine of animals with established infection [46]. Nevertheless, there may be other factors such as the possibility of suppression of viral transcription via release of anti-viral cytokines or possibly even direct killing of the infected cells by a CD4+ T cell-dependent response that contribute to viral control. Lastly, we cannot exclude the possibility that the antibody-based CD8+ T cell depletion was not sufficiently profound (although depletion was >95% as seem in S1 Fig) such that a small fraction remained sufficient to mediate clearance.

Our studies also highlight the contribution of CD4+ T cells in viral control since results show the greatest persistence of virus during asymptomatic infection and viral reactivation (Figs 3 and 4). CD4+ T cells may provide help for the induction of MusPV1-specific CD8+ T cells and/or the induction of L1-specific neutralizing antibodies, and possibly more direct effects. Interestingly, CD4+ depleted mice, which may have partial CD8+ T cell function and are unable to produce neutralizing antibody, still effectively controlled MusPV1 disease (Table 1 and S7 Fig). However, the MusPV1 infection was not cleared and persisted in the lower epithelia cell layers in small patches. Importantly, such infection either has the potential to remain transcriptionally inactive and below the level of detection by simple swabbing. Alternatively its replication is limited and continuously controlled by the remaining subsets of the immune system.

Preclinical studies in C57BL/6 mice demonstrate that HPV16 E6 and E7-specific CD8+ T cell responses and associated anti-tumor immunity elicited by DNA vaccination are significantly enhanced when the antigen is linked to CRT. This reflects improved antigen presentation through the MHC class I pathway [37,38,48] and can occur even in the absence of CD4+ T cell help. An ongoing trial is examining the safety and immunogenicity of the pNGLV4a-CRT/E7 DNA construct in women with high grade CIN (NCT00988559).

Using the same vaccine strategy for MusPV1 antigens, we show that vaccination with CRT/mE6 and CRT/mE7 DNA induced specific CD8+ T cell responses in C57BL/6 mice (Fig 5). In this model a major epitope for mE6 was defined as residues 90–99, while a weaker mE7 epitope was mapped to residues 69–77 (Fig 5 and S5 Fig). Vaccination utilizing DNA encoding CRT fused in tandem to mE6, mE7 and MusPV1 L2 11–200 also was able to induce a potent mE6 aa90-99 -specific CD8+ T cell response in C57BL/6. However, no mE7 response was detected (Fig 5C) suggesting that the E6 response is dominant. In contrast, for the HPV16 version of this DNA vaccine consisting of HPV E6, E7 and L2, the dominant response is to E7 in C57BL/6 mice [38]. Further, CD8+ T cell activation to produce IFNγ for the splenocytes taken from unvaccinated C57BL/6 mice that had spontaneously cleared papilloma detected a T cell response only to mE6 90–99 (S5 Fig). Taken together, these results suggest that in the C57BL/6 background, mE6 90–99 is the immune-dominant and natural epitope and a relevant correlate of antiviral immunity. Interestingly, our results are analogous to other findings that papillomavirus E6 being the dominant antigen in natural host responses in different species such as rabbits and humans [49,50]. By contrast, neither CD4+ nor CD8+ T cell responses to mE6 or mE7 in BALB/c were detected after DNA vaccination despite several attempts. Given that papilloma regression was observed in this strain and clearance is T cell mediated (Fig 2), the failure to detect a mE6 or mE7 T cell immune response in BALB/c is either due to technical issues in peptide mapping design or the therapeutic response is directed to other viral proteins. Indeed several studies in animals and patients have suggested that E1 and E2 are potential rejection antigens [51]. Taken together, our results suggest that it may be beneficial to vaccinate against more than a single viral antigen to elicit therapeutic responses in a greater proportion of patients, but responses to a single epitope can dominate.

Adoptive transfer of MusPV1 E6-specific CD8^+^ T cells into immunodeficient mice protected from and treated papillomatosis, demonstrating direct role of systemic CD8 T cells in controlling MusPV1. This supports their capacity to find, infiltrate, proliferate and control disease, although low levels of virus remained detectable in certain areas of the tail sections at 10 weeks after treatment of mice with a high disease burden (Fig 7C). It is not clear if with further time, these viral reservoirs would have been eliminated. These observations (Figs 6D and 7E) suggest that that neither mucosal vaccination nor local application of adjuvant is required for effective homing, although they might be beneficial.

Of relevance to clinical trial design, our therapeutic investigations using MusPV1 show that a substantial length of time is required to clear established papilloma and that even following lesion regression, viral reservoirs may remain (Fig 7A–7C). Consistent with the notion that greater disease burden is associated with a poorer outcome of immunotherapy, the therapeutic response was more effective against persistent infection in the absence of established disease (Fig 6). Given the increasing use of HPV testing for screening, our results suggest the potential for therapeutic vaccination against persistent infection to prevent the onset of high grade neoplasia. Further, it was recently reported that complete regression of metastatic cervical cancer occurred in 2/9 patients upon systemic administration of a single dose of HPV-targeted tumor-infiltrating T cells [52]. This is consistent with our adoptive T cell study to treat MusPV1-associated papillomas, although this approach is not appropriate for the treatment of premalignant disease in patients.

Our studies were consistent with others showing common inbred strains of mice fully control MusPV1 infection [31,32]. However, for the first time, we show that challenge of the outbred SKH-1 mouse strain leads to the diverse outcomes, as seen in HPV-infected patients (Fig 1); a subset of the outbred SKH-1 mice appear genetically susceptible and will develop persistent infections, whereas the remainder will clear their infections in time or do not develop clinically-apparent lesions. An understanding of the genetic factors in mice driving these different outcomes might be applied to identify those patients who will clear an HPV infection without intervention, and those needing active treatment. Furthermore, while challenge of inbred mice with syngeneic tumor models, e.g. TC-1 and C3, provides critical mechanistic information, studies in outbred SKH-1 mice with MusPV1 may exhibit a range of clinical outcomes that could be more predictive of clinical studies of vaccines and other immunologic interventions. Indeed, these findings in outbred SKH-1 mice clearly demonstrate the importance of genetic background in the outcomes of MusPV1 challenge, possibly reflecting individual differences histocompatability, innate immunity and/or susceptibility to skin carcinogenesis. The tractability of mouse genetics will greatly facilitate further mechanistic analyses.

## Materials and Methods

### Mice & Ethics Statement

6–8 weeks old female C57BL/6J (strain code: 000664), BALB/cJ (strain code: 000651), Rag1 Knock-out (B6.129S7-Rag1^tm1Mom^/J), CD4- Knockout (B6.129S2-Cd4 ^tm1Mak^/J) and CD40 ligand knockout (B6.129S2-Cd40lg ^tm1Imx^/J) were purchased from Jackson Laboratories Inc. Immunodeficient athymic Nude mice (Athymic NCr-nu/nu), SCID mice (NCI SCID/Ncr, BALB/c background) and immuno-competent SKH-1 elite mice (Crl:SKH1-Hr^hr^) were obtained from Charles River, Inc. IFNAR knockout mice were a kind gift from Dr. G. Cheng (University of California, Los Angeles, CA) All animal studies were carried out in accordance with the recommendations in the Guide for the Care and Use of Laboratory Animals of the National Institutes of Health and with the prior approval of the Animal Care and Use Committee of Johns Hopkins University (MO12M223).

### Cell Culture

293^Kb^, 293^Db^, and 293^KbDb^ human embryonic kidney 293 cell lines expressing the murine MHC class I alleles D^b^ and K^b^ or both respectively (all kind gifts from Dr J.C. Yang, NCI, NIH, Bethesda, MD) and CT-26 (ATCC CRL-2638) were maintained in RPMI medium supplemented with 10% fetal bovine serum, 100U penicillin and streptomycin, 1X Non-essential amino acids, 1mM Sodium Pyruvate (Gibco, Life Technologies, Grand Island NY). 293TT cells (kindly provided by Dr. J.T. Schiller, NCI, NIH, Bethesda, MD, see http://home.ccr.cancer.gov/Lco/293TT.htm) were maintained in Dulbecco's modified Eagle medium (DMEM) with 10% fetal bovine serum, 100U penicillin and streptomycin, 1X Non-essential amino acids, 1mM Sodium Pyruvate (Gibco, Life Technologies, Grand Island NY). Lentivirus containing MusPV1 *E6* and *GFP* was prepared and used to infect TC-1 cells. Fluorescent detection of GFP expression was used as a surrogate marker for MusPV E6.

### MusPV1 Genomic DNA and MusPV1 Viral Gene DNA Constructs

pAsylum-MusPV1 and pShell vector encoding codon optimized MusPV1 *L1* and *L2* were kindly provided by Chris Buck (NCI). MusPV1 *E6*, *E7* and *E6E7L2* were codon-optimized and synthesized directly (Biobasic) and inserted after human calreticulin (CRT) between the *EcoR*I and *Not*I sites of pNGLV4a. The MusPV-*E1/E2/E4* genes were PCR amplified from the MusPV1 genome using primers in S1 Table and cloned between the *EcoR*I and *Not*I sites of pNGLV4a. All plasmid constructs were confirmed by DNA sequencing.

### Quantification of MusPV Genomic DNA and E1^E4 Transcript

Briefly, DNA from mice tails was collected via swabbing the challenge site with buccal swabs and extracted as described in http://home.ccr.cancer.gov/lco/VirionExtraction.htm. PCR reaction assays were performed with primers forward 5’-GGTCAAAAGGGCAGCGTCTA-3’, reverse 5’-TGCTTCCCCTCTTCCGTTTT-3’ and were run using SsoFast EvaGreen Supermixes (Bio-Rad) according to the manufacturer’s protocol. Primers were assessed for specificity and sensitivity (S3 Fig). Analysis of MusPV E1^E4 mRNA transcript by reverse transcriptase quantitative PCR (RT-qPCR) was performed as described in [33]. Briefly, mouse tail tissue specimens were flash frozen and stored at -80°C. Tissues were disrupted using a frozen mortar and pestle and homogenized with QIAshredders (Qiagen). RNA for qRT-PCR was extracted using RNeasy Mini kit (Qiagen). cDNA was generated using iScript Advanced cDNA Synthesis Kit (Bio-Rad) and run using TaqMan Gene Expression Master Mix (Applied Biosystems). Primer sequences were E1^E4-forward, 5′-CATTCGAGTCACTGCTTCTGC-3′; E1^E4-reverse, 5′-GATGCAGGTTTGTCGTTCTCC-3′; E1^E4-probe, 5′-6-carboxyfluorescein (FAM)-TGGAAAACGATAAAGCTCCTCCTCAGCC-6-carboxytetramethylrhodamine (TAMRA)-3′. Beta-actin primers (Life Technologies) was used to normalize DNA collection. Triplicate reactions were performed for each primer-probe set.

### Production of MusPV1 Virions and Experimental Challenge

Generation of infectious mouse papilloma virions and propagation of the virus using nude mice were performed as described in [53]. Experimental challenge of mice with MusPV1 virions (with >~10^12^ viral genome equivalents (VGE)) was done using a rotary device (Dremel Drill) as described previously in [33]. In all challenge studies, a group of n = 5 nude or SCID mice was challenge in tandem to act as a technical positive control to ensure that the experimental in vivo challenge was successful.

### Chromogenic In Situ Hybridization

Custom RNA in situ hybridization probes were prepared by the manufacturer (Advanced Cell Diagnostics, Inc.) to detect full-length *E6*/*E7* mRNA sequence of MusPV1. RNAscope assays were performed using the RNAscope 2.0 FFPE Brown Reagent kit according to the manufacturer’s instructions. Briefly, formalin-fixed, paraffin-embedded tissues sections (FFPE) mouse tail sections were pretreated with heat and protease prior to hybridization with probe. To ensure RNA integrity and assay procedure, adjacent sections were also hybridized with a probe to the endogenous housekeeping gene *ubiquitin*. After washing, an HRP-based amplification system was then used to detect the target probes followed by color development with DAB.

### Immunohistochemistry

CD3 was stained by following PowerVision Poly-HRP IHC Detection system protocol (Leica Biosystem). Briefly, FFPE sections were deparaffinized in xylene, followed by dehydration in graded ethanol. Antigen retrieval was performed by steaming specimens at 100°C for 20 min in Target Retrieval Solution (Dako) and subsequently washed in Tris-buffered saline with Tween 20 (TBST, 0.05% Tween 20). Endogenous peroxidase was blocked, by treatment of slides with Dual Endogenous Enzyme-Blocking Reagent (Dako) for 5 min at room temperature. Sections were covered with rabbit monoclonal CD3 primary antibody (ThermoFisher, RM-9107, 1:300) diluted with Antibody Dilution Buffer (ChemMate) and then incubated at room temperature for 45 min. Slides were then washed with TBST, followed by incubation with PowerVision Poly-HRP Anti-Rabbit IgG for 30 min at room temperature. After three washes in TBST, sections were treated with DAB chromogen (3, 3 '-diaminobenzidine tetrahydrochloride; Sigma) for 20 min in the dark. Sections were counterstained with Mayer’s hematoxylin (Dako), dehydrated with ethanol and xylene, and mounted permanently.

### Peptides

A panel of 20mer peptides, each overlapping by 15 amino acids, were generated by Genscript, Inc, (New Jersey) at a purity of ≥70% for both MusPV E6 (25 peptides) and E7 (20 peptides). These 20mers were then pooled into libraries of 5 peptides covering for E6 amino acids 1–40, 25–65, 50–90, 75–115 and 100–140, and for E7 1–40, 25–65, 50–90, and 75–110. 9mer peptides (overlapping by one amino acid) for E6 amino acids 89–104 and E7 66–80 were synthesized to further define their CD8+ T cell epitopes.

### DNA Vaccination via Electroporation

C57BL/6 or BALB/c mice were injected intra-muscularly (hind leg thigh muscle) with 15μg of DNA in 30 μL PBS. Subsequently, a pair of electrode needles was inserted into the muscle area surrounding the DNA injection site. Electrical pulses were delivered using the BTX electroporation generator (ECM830, BTX Harvard Apparatus, Holliston, MA). 8 pulses of 106 V were delivered with a 20 ms pulse at 200 ms intervals.

### Intracellular Cytokine Staining and Flow Cytometry Analysis

Cell lines that expressed the relevant murine MHC class 1 alleles were first seeded at 5x10^5^ cells/well/mL in a 24 well plate before being transfected with the relevant expression vectors. After 48 h, these cells were incubated with 10^7^ splenocytes from either vaccinated or infected mice in the presence of Golgi plug for 12 h, and then collected for cell staining and flow cytometry analysis. For epitope mapping, 10^7^ splenocytes from CRT/mE6, CRT/mE7 or CRT/mE6E7L2 immunized mice were incubated with the peptide libraries of MusPV1 E6 or E7, or later with specific 20mers or 9mers in the presence of Golgi plug for 12 h. The cells were collected for cell staining and flow cytometry analysis. To further characterize the MHC restriction background for MusPVE6 or E7 in the C57BL/6 background, 293^Kb^ or 293^Db^ were pulsed with peptide and co-cultured with 10^7^ splenocytes from either CRT-MusPV-E6 or CRT-MusPV-E7 DNA immunized mice in the presence of Golgi plug for 12 h before being collected for intra-cellular staining and flow cytometry. Intracellular cytokine staining and flow cytometry were performed as previously described in [37].

### In Vivo T Cell Depletion Experiments

In vivo T cell depletions were performed as described previously in [54]. Briefly, mice were treated i.p. with 100μg (1 μg/μL) depletion monoclonal antibodies for CD4 (clone GK 1.5), CD8 (clone 2.43) or CD3 (clone 145-2C11) (BioXcell). Depletions were initiated 1 week prior to MusPV1 virion challenge. Depletion was confirmed using flow cytometry (S1 Fig). Cages were also changed every week to minimize contamination from remaining inoculum when depletion studies were being maintained.

### Establishment of Murine MusPV1 E6-specific CD8+ T Cell Line

C57BL/6 mice were vaccinated with 25 μg of pcDNA3-CRT/mE6 via intramuscular injection followed by electroporation. Splenocytes were stimulated with irradiated TC-1/MusPV1 E6 cells and murine IL-2 (20 IU/mL). The cells were re-stimulated once a week in the presence of murine IL-2. The specificity of the CD8+ T cells was determined by stimulation with MusPV E6 peptide in the presence of GolgiPlug and followed by CD8 and IFN-γ intracellular staining.

### Sequence Information

Sequence of the MusPV1 construct in pAsylum and individual proteins can be found at http://home.ccr.cancer.gov/Lco/pMusPV.txt and the codon-optimized MusPV1 L1/L2 construct at http://home.ccr.cancer.gov/Lco/pMusheLL.txt.

## Supporting Information

S1 Fig (Related to Table 1). Representative figure showing the CD4+ and CD8+ T cell profiles of mice without prior T cell depletion (A) or mice that went under CD4+ T cell depletion (B), CD8+ T-cell depletion (C) and CD3+ T cell depletion (D). Only CD3+ T cell depletion allows the growth of papillomas on both C57BL/6 mice (E) and BALB/c (F) therefore showing control of papillomavirus is due to T-cell immunity in both strains.
(TIF)

S2 Fig Restoration of T cell immunity results in wart regression (Related to Fig 2). (A) Papillomas appeared on the tails of nine BALB/c mice following continuous T cell depletion using CD3 antibody at 5-weeks post infection. At this time, the mice were divided; 5 continued undergoing depletion with CD3 antibody for another 5 weeks resulting is more obvious papillomas (B) whereas in the remaining 4 mice the depletion was stopped and wart regression was seen in all cases (C).
(TIF)

S3 Fig PCR detection of MusPV1 DNA. (A) A primer set was tested to assess sensitivity via qPCR with varying quantities of re-ligated MusPV genome to create a 10-fold dilution standard curve (range 5ng/μL to 5X10^-9^ ng/μL). PCR efficiency was calculated to be approximately 110% (correlation co-efficient = 0.998, slope = -3.099) and the limit of detection was 6 viral genomic copies. (B) Representative agarose gel results showing the specificity of detecting MusPV1 genomic DNA via PCR (lane 5) and tail swabs of mice that were negative for papilloma (lanes 2 and 3). A water control (for technical validation) was also included (lane 6) and molecular weight markers (lanes 1 and 4). (C) Tail swabs from MusPV1 virus challenged BALB/c mice that underwent no depletion (wildtype control), CD4 T cell depletion (CD4 dep), CD8 T cell depletion (CD8 dep) were tested in conjunction with nude mice as a positive control and uninfected mice (as negative control).
(TIF)

S4 Fig Determining if a CD8+ T cell response exists against MusPV1 E6 or E7 via intracellular cytokine staining and flow cytometry analysis in C57BL/6 or BALB/c mice. Representative flow cytometry results after intracellular cytokine staining of splenocytes for interferon-γ and CD8 after harvest from hCRTmE6- or hCRTmE7-vaccinated mice and co-culture with either 293^DBKB^ or CT26 cells (which over-express the murine MHC class I of C57BL/6 and BALB/c respectively) and had been transfected with expression vectors for either MusPV1 E6 or E7. A mE6 specific CD8+ T-cell response was detected in hCRTmE6 vaccinated C57BL/6 mice but no mE7 specific CD8+ T-cell response was detected in hCRTmE7 C57BL/6 vaccinated mice (A). Neither mE6 nor mE7-specific CD8+ T cell responses were detected in hCRTmE6 or hCRTmE7 vaccinated BALB/c mice respectively (B). To address potential sensitivity issues in the BALB/c studies, the CD8+ T cell activation assays were repeated using 20mer over-lapping peptide libraries derived from MusPV1 E6 and E7 amino acid sequences for stimulation. No mE6 or mE7 specific CD8+ T-cell responses were detected (C).
(TIF)

S5 Fig Determining the CD8+ T cell epitope of MusPV1 E7 and its MHC class I binding restriction via intracellular cytokine staining and flow cytometry analysis. Bar graph of flow cytometry results after intracellular cytokine staining of splenocytes for interferon-γ and CD8 after harvest from CRTmE7-vaccinated mice and stimulation with mE7 peptide library pools (A). Bar graph summarizing flow cytometry data after intracellular cytokine staining of splenocytes for interferon-γ and CD8 after harvest from CRTmE7-vaccinated mice and stimulated with candidate 9mer peptides to map the immune-dominant MHC class I epitopes of MusPV1 mE7 (B). Bar graph showing flow cytometry data for percentages of interferon-γ expressing mE7 specific CD8+ T cells after co-incubation with varying amounts of 293 cells expressing either the Murine MHC class I molecule H-2K^b^ (293-K^b^ or 293-K^d^) that were pulsed with the MusPV E7 immunodominant peptide, VLRFIIVTG to determine the MHC restriction. The results show that mE7 is H2-K^b^-restricted (C).
(TIF)

S6 Fig Natural response to MusPV1-induced papilloma regression in C57/BL6 is mE6 dominant. CD3 antibody depletion of a group of 5 C57/BL6 and 5 BALB/c bearing florid MusPV1 papilloma was stopped. Once the papilloma were completely regressed, splenocytes were harvested, pooled and incubated with either 293^DbKb^ cells or CT27 cells which were transfected with expression vectors for either hCRT-alone (con), or hCRT-linked to either MusPV1 E1, E2, E4, E6, E7, L1 or L2 respectively. The splenocytes were also stimulated directly using the MusPV1 E6 a.a. 90–99 (KNIVFVTVR) and E7 a.a. 69–77 (VLRFIIVTG) peptide (PEP) epitopes. An mE6 specific CD8 T cell response could be detected in the splenocytes of C57BL/6 mice that had spontaneously cleared their papilloma only in the mE6 peptide stimulated cells. However, no immune responses was detected in the BALB/c mice.
(TIF)

S7 Fig Establishment of a MusPV1 E6-specific murine CD8+ T cell line. TC-1 cells were infected with lentivirus co-expressing MusPV1 E6 and GFP. GFP/mE6 expressing TC-1 cells were analyzed with flow cytometry analysis (A). TC-1 cells expressing MusPV1 E6 are able to activate MusPV1 E6-specific CD8+ T cells after DNA vaccination. C57BL/6 mice were vaccinated with pcDNA3-CRT/mE6 via intramuscular injection followed by electroporation. Splenocytes were prepared and stimulated with either MusPV1 E6 peptide, or irradiated TC-1 expressing GFP/mE6 or HPV11E6E7GFP (control) overnight in the presence of GolgiPlug. The cells were stained with anti-mouse CD8 antibody. After permeabilization and fixation, the cells were stained anti-mouse IFN-γ antibody. The cells were acquired with FACSCalibur flow cytometer and analyzed with CellQuest Pro software (B). Establishment of murine MusPV1 E6-specific CD8+ T cell line. pcDNA3-CRT/mE6 vaccinated C57BL/6 mouse splenocytes were stimulated with irradiated TC-1/mE6 cells at the presence of murine IL-2 (20 IU/ml) once a week for four weeks. The cells were stimulated with MusPV1 E6 peptide in the presence of GolgiPlug overnight. IFN-γ intracellular staining was then performed to determine the specificity of the CD8+ T cells (C).
(TIF)

S8 Fig CD4+ T cells are important for induction of MusPV1 neutralizing antibody response. Mice that were first depleted of CD3+, CD4+ or CD8+ T cell subsets were challenged with MusPV1 virus and assessed for their MusPV1 neutralizing antibody titers 5 weeks post infection (A). MusPV1 infected mice that were first depleted using CD3, CD4 or CD8-specific antibody for 5 weeks, were switched to 10 more weeks of CD3 antibody treatment to fully deplete their T cell population (B).
(TIF)

S1 Table Design of primers used to amplify MusPV1 genes. (DOCX)
