# Supplementary figures and images for: Immunologic Control of Mus musculus Papillomavirus Type 1
Source: PLoS Pathog. 2015 Oct 23;11(10):e1005243. doi: 10.1371/journal.ppat.1005243 (PMC4619818; doi:10.1371/journal.ppat.1005243)

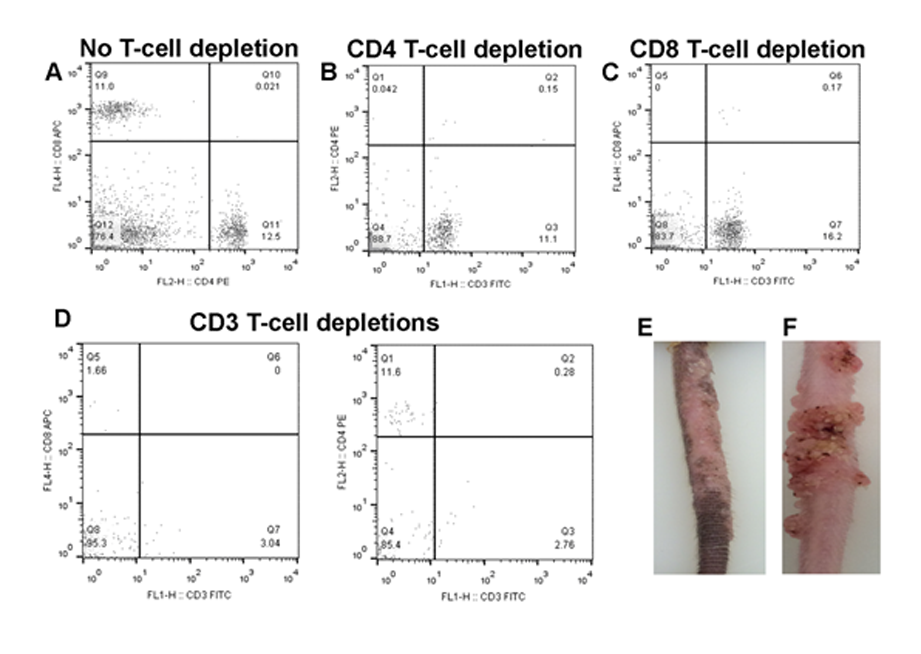

Supplement: S1 Fig — Representative figure showing the CD4+ and CD8+ T cell profiles of mice without prior T cell depletion (A) or mice that went under CD4+ T cell depletion (B), CD8+ T-cell depletion (C) and CD3+ T cell depletion (D). Only CD3+ T cell depletion allows the growth of papillomas on both C57BL/6 mice (E) and BALB/c (F) therefore showing control of papillomavirus is due to T-cell immunity in both strains. (TIF) [file ppat.1005243.s001.tif]

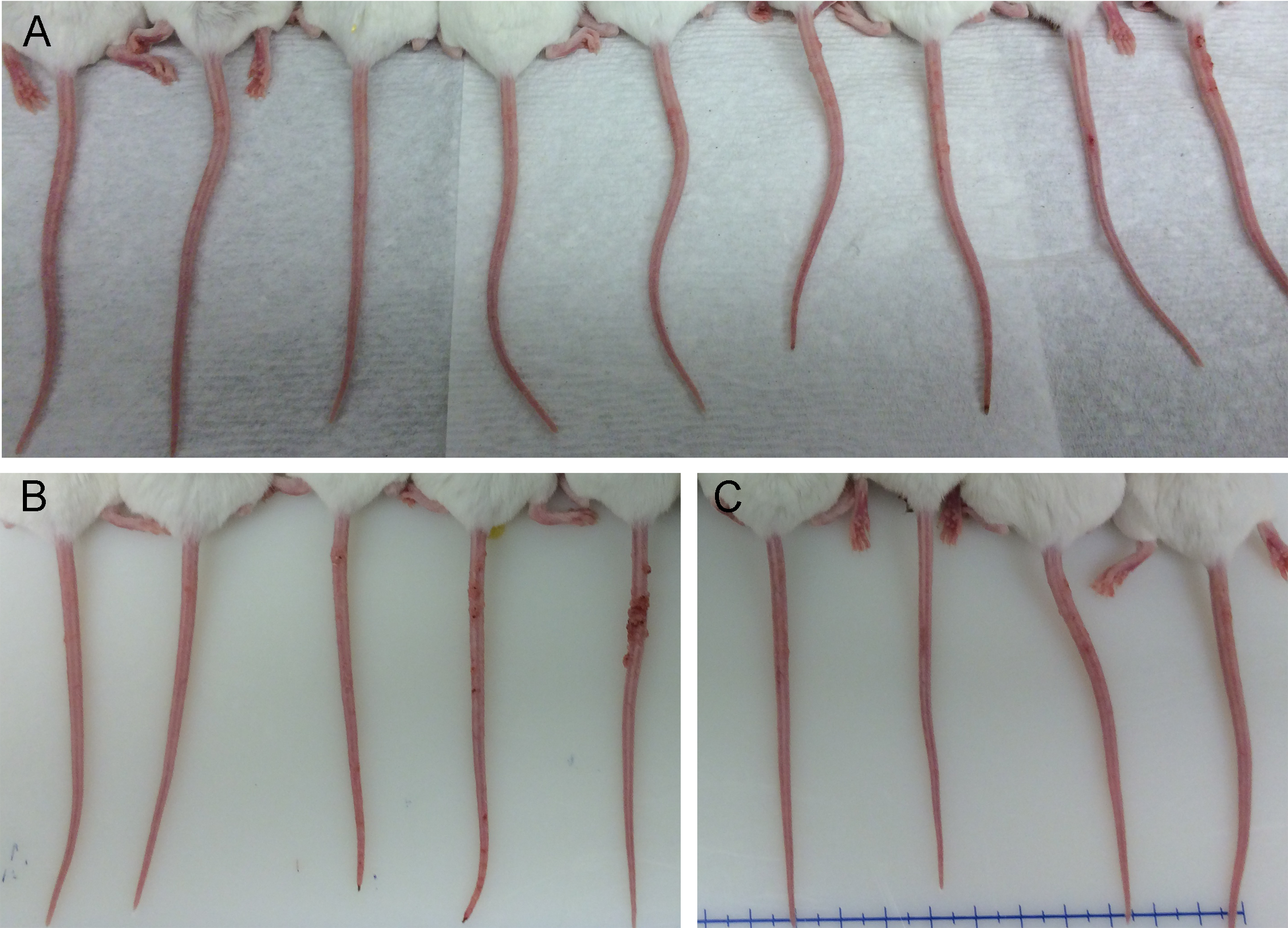

Supplement: S2 Fig — (A) Papillomas appeared on the tails of nine BALB/c mice following continuous T cell depletion using CD3 antibody at 5-weeks post infection. At this time, the mice were divided; 5 continued undergoing depletion with CD3 antibody for another 5 weeks resulting is more obvious papillomas (B) whereas in the remaining 4 mice the depletion was stopped and wart regression was seen in all cases (C). (TIF) [file ppat.1005243.s002.tif]

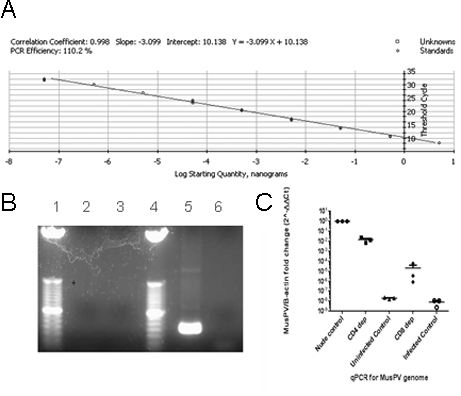

Supplement: S3 Fig — (A) A primer set was tested to assess sensitivity via qPCR with varying quantities of re-ligated MusPV genome to create a 10-fold dilution standard curve (range 5ng/μL to 5X10-9 ng/μL). PCR efficiency was calculated to be approximately 110% (correlation co-efficient = 0.998, slope = -3.099) and the limit of detection was 6 viral genomic copies. (B) Representative agarose gel results showing the specificity of detecting MusPV1 genomic DNA via PCR (lane 5) and tail swabs of mice that were negative for papilloma (lanes 2 and 3). A water control (for technical validation) was also included (lane 6) and molecular weight markers (lanes 1 and 4). (C) Tail swabs from MusPV1 virus challenged BALB/c mice that underwent no depletion (wildtype control), CD4 T cell depletion (CD4 dep), CD8 T cell depletion (CD8 dep) were tested in conjunction with nude mice as a positive control and uninfected mice (as negative control). (TIF) [file ppat.1005243.s003.tif]

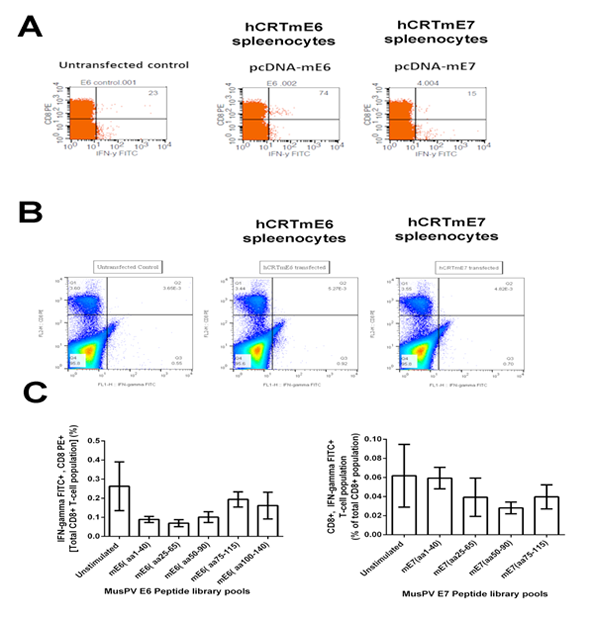

Supplement: S4 Fig — Representative flow cytometry results after intracellular cytokine staining of splenocytes for interferon-γ and CD8 after harvest from hCRTmE6- or hCRTmE7-vaccinated mice and co-culture with either 293DBKB or CT26 cells (which over-express the murine MHC class I of C57BL/6 and BALB/c respectively) and had been transfected with expression vectors for either MusPV1 E6 or E7. A mE6 specific CD8+ T-cell response was detected in hCRTmE6 vaccinated C57BL/6 mice but no mE7 specific CD8+ T-cell response was detected in hCRTmE7 C57BL/6 vaccinated mice (A). Neither mE6 nor mE7-specific CD8+ T cell responses were detected in hCRTmE6 or hCRTmE7 vaccinated BALB/c mice respectively (B). To address potential sensitivity issues in the BALB/c studies, the CD8+ T cell activation assays were repeated using 20mer over-lapping peptide libraries derived from MusPV1 E6 and E7 amino acid sequences for stimulation. No mE6 or mE7 specific CD8+ T-cell responses were detected (C). (TIF) [file ppat.1005243.s004.tif]

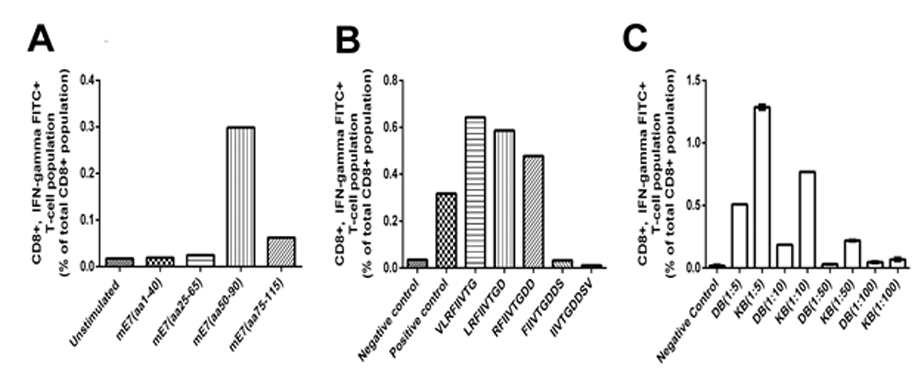

Supplement: S5 Fig — Bar graph of flow cytometry results after intracellular cytokine staining of splenocytes for interferon-γ and CD8 after harvest from CRTmE7-vaccinated mice and stimulation with mE7 peptide library pools (A). Bar graph summarizing flow cytometry data after intracellular cytokine staining of splenocytes for interferon-γ and CD8 after harvest from CRTmE7-vaccinated mice and stimulated with candidate 9mer peptides to map the immune-dominant MHC class I epitopes of MusPV1 mE7 (B). Bar graph showing flow cytometry data for percentages of interferon-γ expressing mE7 specific CD8+ T cells after co-incubation with varying amounts of 293 cells expressing either the Murine MHC class I molecule H-2Kb (293-Kb or 293-Kd) that were pulsed with the MusPV E7 immunodominant peptide, VLRFIIVTG to determine the MHC restriction. The results show that mE7 is H2-Kb-restricted (C). (TIF) [file ppat.1005243.s005.tif]

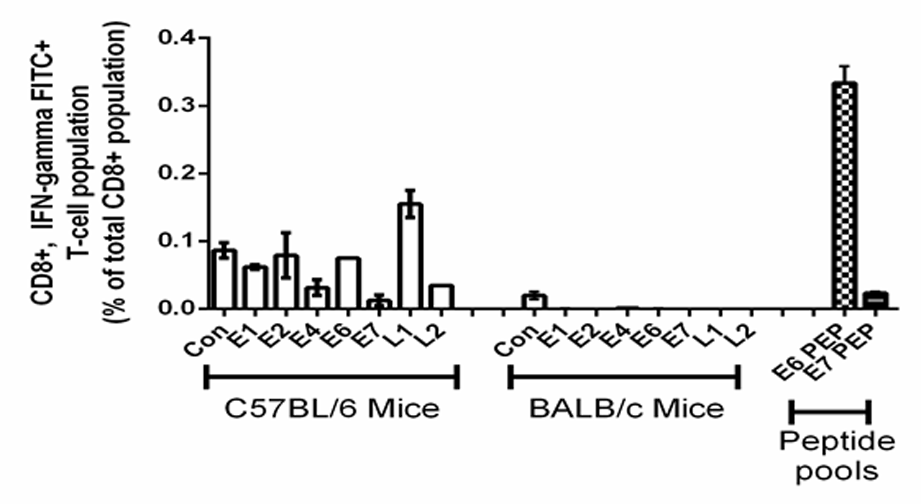

Supplement: S6 Fig — CD3 antibody depletion of a group of 5 C57/BL6 and 5 BALB/c bearing florid MusPV1 papilloma was stopped. Once the papilloma were completely regressed, splenocytes were harvested, pooled and incubated with either 293DbKb cells or CT27 cells which were transfected with expression vectors for either hCRT-alone (con), or hCRT-linked to either MusPV1 E1, E2, E4, E6, E7, L1 or L2 respectively. The splenocytes were also stimulated directly using the MusPV1 E6 a.a. 90–99 (KNIVFVTVR) and E7 a.a. 69–77 (VLRFIIVTG) peptide (PEP) epitopes. An mE6 specific CD8 T cell response could be detected in the splenocytes of C57BL/6 mice that had spontaneously cleared their papilloma only in the mE6 peptide stimulated cells. However, no immune responses was detected in the BALB/c mice. (TIF) [file ppat.1005243.s006.tif]

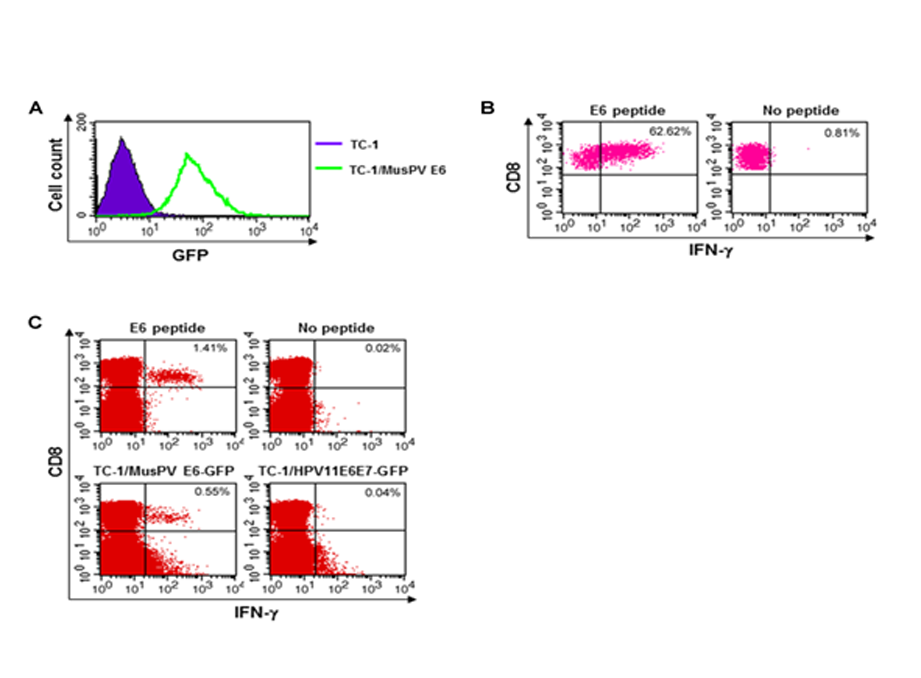

Supplement: S7 Fig — TC-1 cells were infected with lentivirus co-expressing MusPV1 E6 and GFP. GFP/mE6 expressing TC-1 cells were analyzed with flow cytometry analysis (A). TC-1 cells expressing MusPV1 E6 are able to activate MusPV1 E6-specific CD8+ T cells after DNA vaccination. C57BL/6 mice were vaccinated with pcDNA3-CRT/mE6 via intramuscular injection followed by electroporation. Splenocytes were prepared and stimulated with either MusPV1 E6 peptide, or irradiated TC-1 expressing GFP/mE6 or HPV11E6E7GFP (control) overnight in the presence of GolgiPlug. The cells were stained with anti-mouse CD8 antibody. After permeabilization and fixation, the cells were stained anti-mouse IFN-γ antibody. The cells were acquired with FACSCalibur flow cytometer and analyzed with CellQuest Pro software (B). Establishment of murine MusPV1 E6-specific CD8+ T cell line. pcDNA3-CRT/mE6 vaccinated C57BL/6 mouse splenocytes were stimulated with irradiated TC-1/mE6 cells at the presence of murine IL-2 (20 IU/ml) once a week for four weeks. The cells were stimulated with MusPV1 E6 peptide in the presence of GolgiPlug overnight. IFN-γ intracellular staining was then performed to determine the specificity of the CD8+ T cells (C). (TIF) [file ppat.1005243.s007.tif]

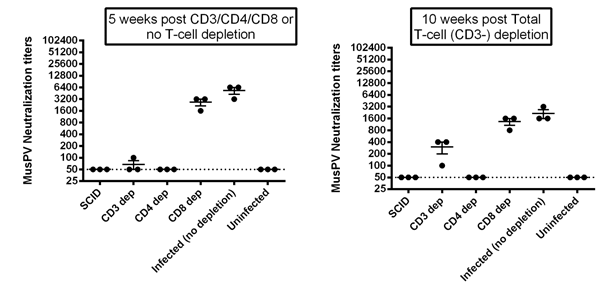

Supplement: S8 Fig — Mice that were first depleted of CD3+, CD4+ or CD8+ T cell subsets were challenged with MusPV1 virus and assessed for their MusPV1 neutralizing antibody titers 5 weeks post infection (A). MusPV1 infected mice that were first depleted using CD3, CD4 or CD8-specific antibody for 5 weeks, were switched to 10 more weeks of CD3 antibody treatment to fully deplete their T cell population (B). (TIF) [file ppat.1005243.s008.tif]
