# Supplementary material for: Immunologic Control of Mus musculus Papillomavirus Type 1
Source: PLoS Pathog. 2015 Oct 23;11(10):e1005243. doi: 10.1371/journal.ppat.1005243 (PMC4619818; doi:10.1371/journal.ppat.1005243)
Supplement: S1 Table — (DOCX) [file ppat.1005243.s009.docx]

**Table S1. Design of primers used to amplify MusPV1 genes**

| MusPV1 protein | Primer Set |
| --- | --- |
| E1 | Forward 5’- GCGCGAATTCATGGAAAACGATAAAGGTACAGGG  Reverse 5’- GAGAGCGGCCGCTTACTGCCTTTCTCGTAAAGG |
| E2 | Forward 5’ -GCGCGAATTCATGAACAGCCTGGAAACACGTTT  Reverse 5’- GAGAGCGGCCGCTCAGAGTCCGTCTAAGAAG |
| E4 | Forward 5’- GCGCGAATTCATGAATCACCCTTGGCTCCGAG  Reverse 5’- GAGAGCGGCCGCTCACAGTCCAGTGAGAATAAT |
| E6 | Forward 5’-GAGAAAGCTTCCACCATGGAGATCGGAAAAGGGTATA  Reverse 5’- GACCGAGAATTCTTATCACAGCAGGGGTTTACAAAAG |
| E7 | Forward 5’- GAGAAAGCTTCCACCATGCAGGGGCCTCTC  Reverse 5’-GACCGAGAATTCTTATCACCTTTTCCCATTCCGCAGATTC |
